# Structural Features of Tight-Junction Proteins

**DOI:** 10.3390/ijms20236020

**Published:** 2019-11-29

**Authors:** Udo Heinemann, Anja Schuetz

**Affiliations:** 1 Macromolecular Structure and Interaction Laboratory, Max Delbrück Center for Molecular Medicine, 13125 Berlin, Germany; 2 Protein Production & Characterization Platform, Max Delbrück Center for Molecular Medicine, 13125 Berlin, Germany

**Keywords:** tight junction, protein structure, protein domain, claudins, occludin, tricellulin, junctional adhesion molecule, *zonula occludens*, MAGUK proteins, PDZ domain

## Abstract

Tight junctions are complex supramolecular entities composed of integral membrane proteins, membrane-associated and soluble cytoplasmic proteins engaging in an intricate and dynamic system of protein–protein interactions. Three-dimensional structures of several tight-junction proteins or their isolated domains have been determined by X-ray crystallography, nuclear magnetic resonance spectroscopy, and cryo-electron microscopy. These structures provide direct insight into molecular interactions that contribute to the formation, integrity, or function of tight junctions. In addition, the known experimental structures have allowed the modeling of ligand-binding events involving tight-junction proteins. Here, we review the published structures of tight-junction proteins. We show that these proteins are composed of a limited set of structural motifs and highlight common types of interactions between tight-junction proteins and their ligands involving these motifs.

## 1. Introduction

A classical paper published more than half a century ago [1] clearly demonstrated that the epithelia of several glands and cavity-forming internal organs of the rat and guinea pig all share characteristic tripartite junctional complexes between adjacent cells. These junctional complexes were found in the epithelia of the stomach, intestine, gall bladder, uterus, oviduct, liver, pancreas, parotid, thyroid, salivary ducts, and kidney. Progressing from the apical to the basal side of the endothelial cell layer, the elements of the junctional complex were characterized as tight junctions (*zonulae occludens*), adherens junctions, and desmosomes. As most apical elements of the junctional complex, tight junctions (TJs) were distinct by the apparent fusion of adjacent cell membranes over variable distances and appeared as a diffuse band of dense cytoplasmic material in the electron microscope. TJs formed a continuous belt-like structure, whereas desmosomes displayed discontinuous button-like structures, and adherens junctions (AJs) were intermediate in appearance. The molecular composition of TJs was revealed in subsequent work by many laboratories, e.g., [2,3,4,5], and shown to include at least 40 different proteins. 

In the pioneering work of Farquhar and Palade, TJs were proposed to function as effective diffusion barriers or seals [1]. The sealing function of TJs contributes to the formation and physiological function of the blood-brain barrier (BBB), which consists of endothelial cells sealed by apical junctional complexes including TJs. Functions of transmembrane TJ proteins at the BBB are well documented [6,7,8]. BBB dysfunction is linked to a number of diseases including multiple sclerosis, stroke, brain tumors, epilepsy, and Alzheimer’s disease [9,10]. 

Here, we review the current literature regarding three-dimensional structures of TJ proteins, their domains and intermolecular interactions. We do not primarily aim at presenting each and every structure in detail, but attempt to distill common structural principles that underlie the architecture of the TJ. We apologize to those authors whose work may not have been covered in this paper for reasons of space and readability.

## 2. Structural Insight into Tight-Junction Proteins, Their Domains, and Interactions

**Figure 1:**
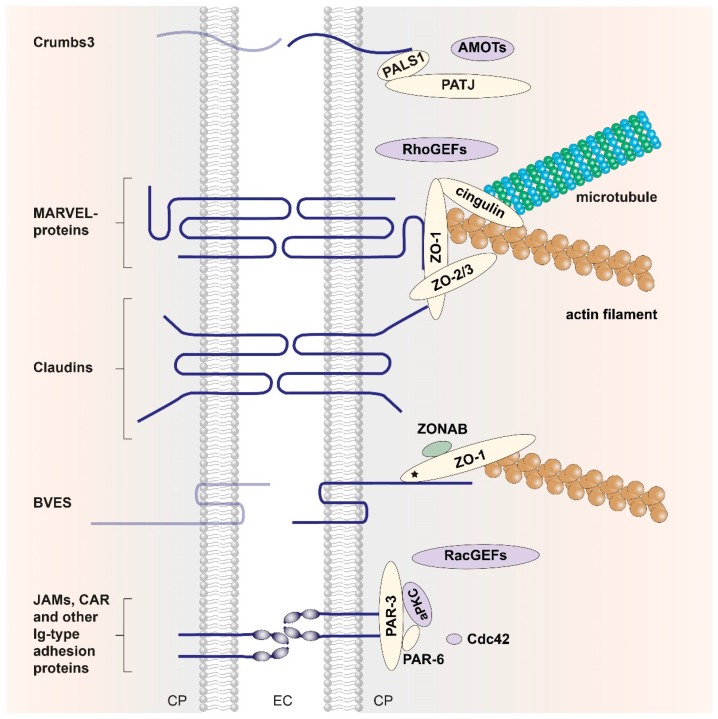
The tight-junction core structure. TM proteins of the TJ (dark blue) interact with a complex cytoplasmic protein network, the cytoplasmic plaque (shown on the right), providing a physical link to the cytoskeleton (microtubules, actin filaments). Cross-membrane interactions between TM proteins are indicated schematically. For some TM proteins (shown in pale blue) there is no direct evidence for a *trans* pairing interaction between TM proteins of opposing cells. The cytoplasmic plaque is composed of scaffolding proteins (yellow ovals) that associate with signaling proteins (purple ovals) and (post)transcriptional regulators (green oval), forming the zonular signalosome [23]. The three major protein complexes located in the cytoplasmic plaque are depicted. Within the ZO complex, ZO proteins are present as homodimers or ZO-1/ZO-2 and ZO-1/ZO-3 heterodimers [24] that directly associate with integral TJ membrane proteins through multiple interactions. The polarity complexes PAR-3/PAR-6/aPKC and Crumbs/PALS1/PATJ are responsible for the development of the apico-basal axis of epithelial cells and act as apical components of TJs. TM proteins: Crumbs homolog 3 (CRB3); MARVEL-domain containing proteins occludin, tricellulin, and MARVEL domain-containing protein 3 (MARVELD3); the claudins; the protein blood vessel epicardial substance (BVES); immunoglobulin (Ig) superfamily members such as junctional adhesion molecules (JAMs) and the coxsackievirus–adenovirus receptor (CAR). Cytoplasmic scaffolding proteins: *Zonula occludens* (ZO) proteins ZO-1, ZO-2, and ZO-3; partitioning defective 3/6 homologs (PAR-3, PAR-6); protein associated with Lin-7 1 (PALS1); PALS1-associated tight junction (PATJ) protein; cytoskeletal linker cingulin. Signaling proteins: Atypical protein kinase C (aPKC); proteins of the angiomotin family (AMOTs) [25]; the small Rho-GTPase Cdc42, and guanine nucleotide exchange factors for the Rho-GTPases RhoA (RhoGEFs, e.g., ARHGEF11 [26]) and Rac1 (RacGEFs, e.g., Tiam-1 [27]), respectively. Transcriptional regulator: ZO-1–associated nucleic acid-binding protein (ZONAB, YBX3 in human) [28]. Figure modified and updated after Zihni et al. [12].

In the most general, birds-eye description, the TJ consists of a set of transmembrane (TM) proteins and the cytoplasmic plaque, a complex network of scaffolding and effector proteins that connects the TM proteins to the actomyosin cytoskeleton of the cell (Figure 1). The TM proteins interact with their extracellular domains in the paracellular space, and the connection to the cytoskeleton inside the cell is structurally as yet uncharacterized [2,3,4,5,11,12]. The *zonula occludens* proteins ZO-1, ZO-2, and ZO-3 and the two mammalian polarity complexes PAR-3/PAR-6/aPKC and Crumbs/ PALS1/PATJ are central players of the cytoplasmic plaque and are described in more detail in this review together with the transmembrane TJ proteins. 

### 2.1. Tight-Junction Transmembrane Proteins

TJ transmembrane proteins contain either one, three, or four TM segments. The Crumbs proteins (CRBs), the junctional adhesion molecules (JAMs), the angulin proteins, and the coxsackievirus–adenovirus receptor (CAR) are representatives of single-span TJ membrane proteins. BVES (blood-vessel epicardial substance, also known as POPDC1 for Popeye domain-containing protein-1) is a TJ-associated protein with three TM regions [13,14]. The claudins and the TAMPs (tight junction-associated MARVEL-domain proteins) occludin (MARVELD1), tricellulin (MARVELD2), and MARVELD3 are tetra-span TM proteins. MARVEL is used as a common acronym for MAL (myelin and lymphocyte) and related proteins for vesicle trafficking and membrane link [15]. Where crystal structures are available, for example the claudin family (Section 2.1.2, [16]), the TM segments were shown to be α-helical. 

#### 2.1.1. Junctional Adhesion Molecules and Other Ig-Like TJ Proteins

**Figure 2:**
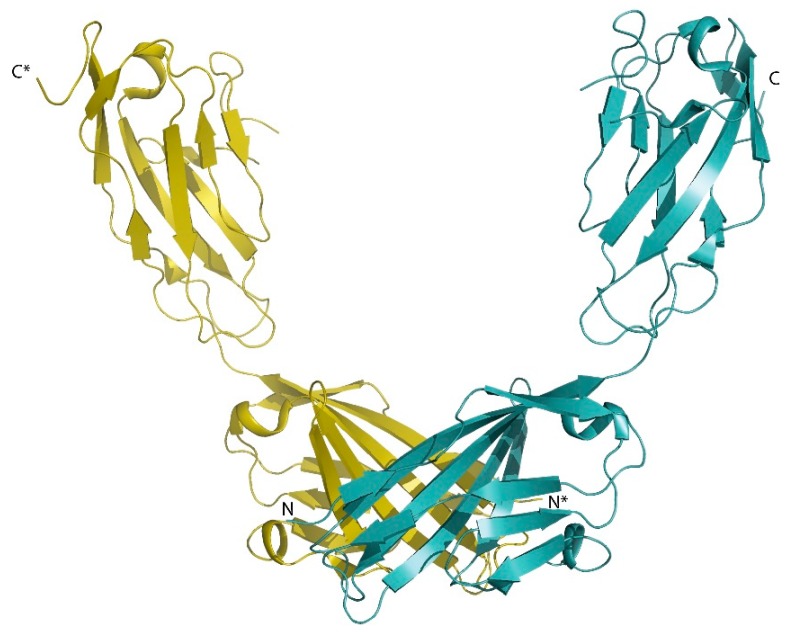
JAM-A dimerization via extracellular Ig domains. Crystal structure of murine JAM-A (PDB entry 1F97) [18]. The dimer is generated by crystallographic two-fold symmetry.

The JAMs are a family of adhesion molecules with immunoglobulin (Ig)-like ectodomains, localized in epithelial and endothelial cells, leukocytes, and myocardial cells [17]. The 2.5-Å crystal structure of the soluble extracellular part of mouse JAM-A provided the first structural insight into a TJ transmembrane protein [18]. In this structure, two Ig domains are connected by a short linker peptide, and a U-shaped dimer is formed by symmetrical interaction of the N-terminal Ig domains (Figure 2). This structure provided the basis for a model of homophilic interactions between the N-domains to explain the adhesive function of JAMs in the TJ. The crystal structure of the extracellular Ig domains of coxsackievirus–adenovirus receptor CAR, another component of the epithelial apical junction complex that is essential for TJ integrity [19], suggests a very similar mode of CAR homodimer formation through symmetrical interaction of its N-terminal Ig domain [20].

The extracellular portions of JAMs serve as viral attachment sites. Reoviruses attach to human cells by binding to cell–surface carbohydrates and the junctional adhesion molecule JAM-A. The crystal structure of reovirus attachment protein σ1 bound to the soluble form of JAM-A shows that σ1 disrupts the native JAM-A dimer to form a heterodimer via the same interface as used in JAM-A homodimers, but with a 1000-fold lower dissociation constant of the σ1/JAM-A heterodimer as compared to the JAM-A homodimer [21]. In cat, infection with calicivirus is initiated by binding of the minor capsid protein VP2 to feline junctional adhesion molecule A (JAM-A). High-resolution cryo-EM structures of VP2 and soluble JAM-A-decorated VP2 show formation of a large portal-like assembly, which is hypothesized to serve as a channel for the transfer of the viral genome [22].

In addition to JAMs and CAR, other single-span Ig-like adhesion molecules such as the endothelial cell-selective adhesion molecule (ESAM), the coxsackievirus and adenovirus receptor-like membrane protein (CLMP), the brain- and testis-specific immunoglobulin superfamily protein (BT-IgSF or IgSF11) [19], and the angulin family of proteins are present at TJs. The latter comprise the proteins LSR (lipolysis-stimulated lipoprotein receptor), ILDR1, and ILDR2 (Ig-like domain-containing receptors) that complement each other at tricellular TJs and co-operate with tricellulin to mediate full barrier function in epithelial sheets [29,30]. Loss of LSR is linked to cell invasion and migration in human cancer cells [31]. To date, however, no structural data are available for any of these proteins.

#### 2.1.2. Claudins

Members of the claudin family are the most abundant TM proteins of the TJ [32]. Claudin genes are expressed across all epithelial tissues, and in all epithelia various different claudins are expressed at the same time [12,33,34,35,36]. Tissue-specific expression of claudin genes has been documented, for example, for claudins in the kidney, inner ear, and eye [33]. 

**Figure 3:**
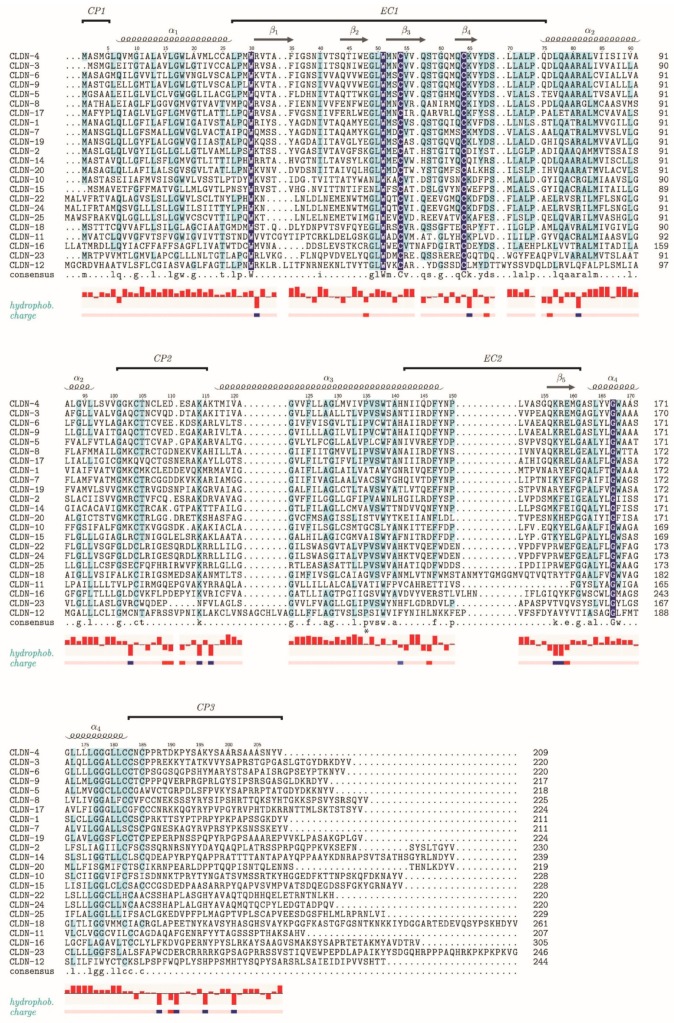
Sequence alignment of human claudins. The Uniprot database [41] lists 23 human claudin sequences belonging to CLDN-1 to CLDN-12 and CLDN-14 to CLDN-25; CLDN-21 is sometimes referred to as CLDN-24. In addition, there are two claudin domain-containing proteins (CLDND1 und CLDND2), which are not included in the alignment. 66 N-terminal residues of CLDN-16 and 46 C-terminal residues of CLDN-23 are omitted from the alignment, because they have no match in any other human claudin sequence. Domain and secondary-structure annotation follows CLDN-4 for which a crystal structure is known in the presence of a bound toxin and loop EC1 is fully ordered [16,42], and the claudin sequences are listed in order of their match with the CLDN-4 sequence. Residues conserved across all human claudins are highlighted on dark blue background and residues conserved in ≥ 50% of the sequences are shown on a light blue background. EC: Extracellular, CP: Cytoplasmic. Conservation of hydrophobicity and charge (blue, positive; red, negative) is indicated at the bottom of the alignment. The asterisk marks a proline residue within α3 of claudin-3, which induces a kink in this helix and probably most other claudins. The amino-acid sequences were aligned using the Clustal Omega server [43], and TEXshade [44] was used for illustration.

At TJs, claudins are arranged to form extended strands by homophilic or heterophilic *cis* pairing within the same membrane or *trans* pairing across membranes [37,38]. In humans, 23 claudins and two claudin-like proteins are currently known (Figure 3). Although clearly homologous, the claudins share only a small number of strictly conserved residues and differ in the lengths of their N- and C-termini and the loops connecting their four TM helices. Highest sequence conservation is observed within the first extracellular loop (ECL1) where a tryptophan and two cysteine residues are strictly conserved in all sequences, suggesting formation of a disulfide bond in this region, which was experimentally verified by crystal structure analysis [16]. Various schemes for grouping claudins have been proposed. Based on sequence conservations, a grouping into classical claudins (1–10, 14, 15, 17, 19) and non-classical claudins (11–13, 16, 18, 20–24) was suggested [39], but the alignment shown in Figure 3 does not clearly support a separation into these groups. Based on function within the TJ, claudins may be grouped according to their barrier or channel forming properties with respect to different solutes [34,35,40]. Claudins 1, 3, 5, 11, 14, and 19, for example, have been characterized as predominantly sealing, whereas claudins 2, 10a, 10b, 15, and 17 were described as predominantly channel forming. For claudins 4, 7, 8, and 16 a sealing or channel-forming function has not been unequivocally determined [34]. Moreover, assignment of these functions to TM proteins of the TJ may be difficult when individual TJ proteins are functionally replaced by paralogs in certain epithelia or in the presence of post-translational modification.

A major breakthrough in TJ research was made in 2014 when the 2.4-Å resolution crystal structure of full-length mouse claudin-15 was reported [45]. As predicted from the sequence, the polypeptide chain was organized into four antiparallel TM helices with the N- and C-termini on the cytoplasmic side. On the extracellular side, a five-stranded up-and-down antiparallel β-sheet was formed by the long ECL1 (strands β1-β4) and the short ECL2 (strand β5, pairing with β1). In this crystal structure, ECL1 is partially disordered, because the loop (v1) connecting strands β1 and β2 is not represented in electron density [45], but it is ordered in the presence of bound ligand (see below). A molecular dynamics study based on the claudin-15 crystal structure [45] suggested that the protein forms a tetrameric channel in which a cage of four aspartate-15 residues acts as a selectivity filter that favors cation flux over anion flux [46]. 

This and other claudin structures are hoped to provide a basis for the targeted disruption of epithelial barriers in the administration of drugs [47]. The subsequently published crystal structure of mouse claudin-3 showed that proline 134 in TM helix α3 induces a bend in this helix, which is alleviated by the corresponding alanine or glycine mutations. A proline residue at this position is present in the majority of human claudin sequences; a helix bend brought about by this residue is likely to modulate the morphology and adhesiveness of TJ strands [48]. Three-dimensional structures of claudins provide the basis for *in silico* modeling of claudin based TJ self-assembly, their barrier and/or channel forming potential [49,50,51]. 

**Figure 4:**
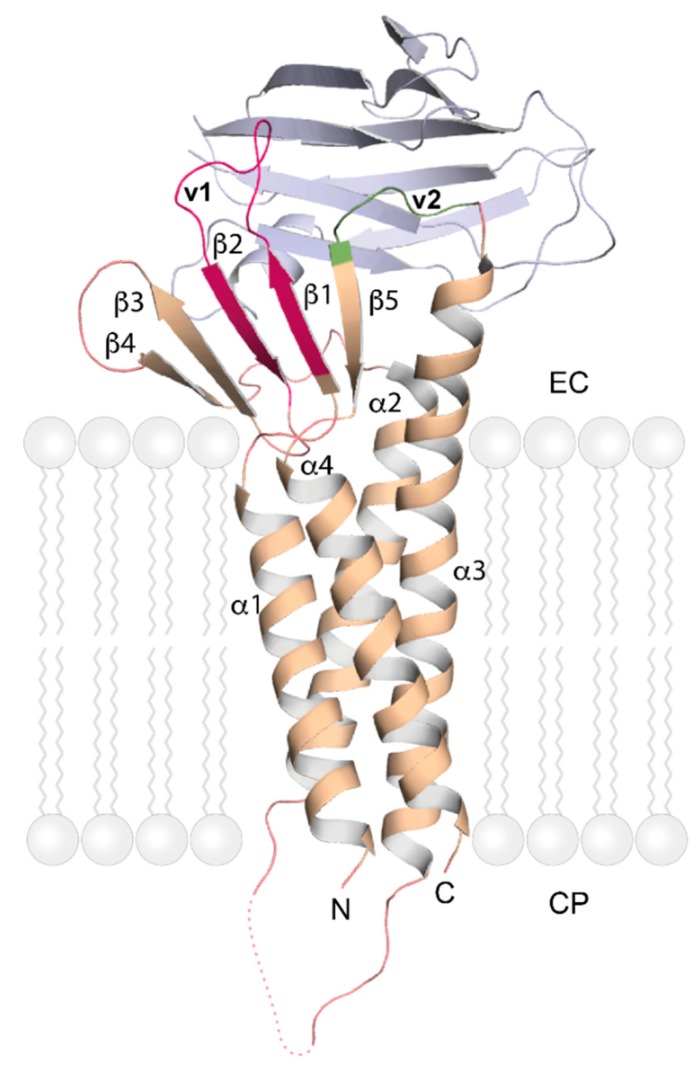
Crystal structure of human claudin-4. Cartoon model of the overall fold of human CLDN-4 (wheat color) in complex with the C-terminal fragment of *Clostridium perfringens* enterotoxin (C-CPE, light blue; PDB entry 5B2G) [16,42]. The extracellular variable regions of CLDN-4 that mediate hetero- and homotypic interactions are highlighted in magenta (v1, comprising β1 and β2) and green (v2, between TM-helix α3 and β5), respectively. The dotted line marks a segment of polypeptide chain not represented in electron density. The stylized lipid molecules indicate the cell membrane and are not part of the experimental structure. EC: Extracellular; CP: Cytoplasmic.

Much as the extracellular Ig domains of the JAMs are attachment sites for viruses, the extracellular loops of claudins serve as landing sites for bacterial toxins such as the *Clostridium perfringens* enterotoxin (CpE). A crystal structure of full-length claudin-19 bound to the soluble, claudin-binding C-terminal fragment of CpE (C-CPE) was determined at 3.7 Å resolution [52]. This structure showed that ligand binding leads to a stabilization of loop v1, which is now ordered, and indicated how C-CPE binding to selected claudins may lead to the disintegration of TJs and increased permeability across epithelial layers. C-CPE appears to bind different claudins with a conserved geometry and to disrupt the lateral interactions of their extracellular parts in the same way [16,52] as suggested by the crystal structure of C-CPE-bound human claudin-4 (Figure 4) [42]. Human claudin-9 (hCLDN-9) is highly expressed in the inner ear, essential for hearing and a high-affinity receptor of CpE. Two recently published 3.2-Å crystal structures of hCLDN-9 bound to C-CPE reveal structural changes in claudin epitopes involved in claudin self-assembly and suggest a mechanism for the disruption of claudin and TJ dissociation by CpE [53].

#### 2.1.3. Occludin

Occludin and the other TAMPs of the TJ, tricellulin, and MARVELD3, share with the claudins the general architecture as tetraspan TM proteins with cytoplasmic N- and C-termini. However, the TAMPs are not homologous with claudins and differ in the length and structure of their cytoplasmic domains and extracellular loops.

**Figure 5:**
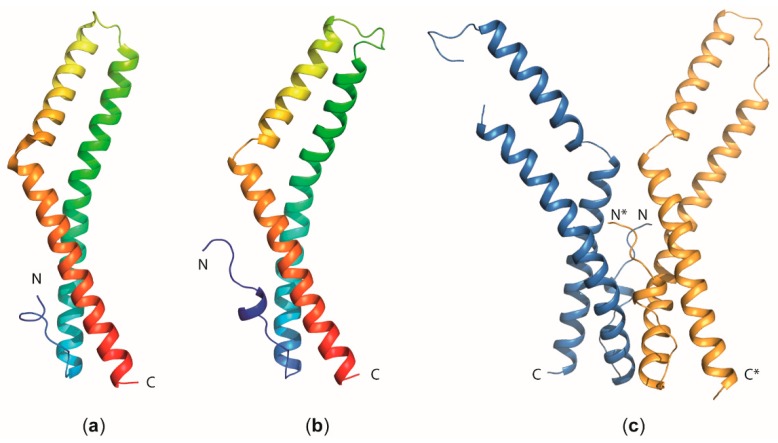
Structural insight into occludin and tricellulin function. Cartoon models of the overall fold of the coiled-coil domain of (**a**) human occludin (PDB entry 1XAW) [58] and (**b**) human tricellulin (PDB entry 5N7K) [71]. The molecules are colored in a gradient ranging from blue at the N-terminus (N) to red at the C-terminus. (**c**) Dimeric arrangement of the tricellulin C-terminal coiled-coil domain observed in the crystal structure [71]. The chain marked with an asterisk (*) corresponds to the second monomer within the dimer.

The occludin cytosolic C-terminus forms a coiled-coil structure, dimerizes, and associates with all three ZO-proteins from the TJ cytoplasmic plaque [24,54,55]. Disulfide formation within the coiled-coil domain was proposed as a mechanism to influence the oligomerization of occludin [56,57]. The 1.45-Å crystal structure of the cytosolic C-terminus of occludin comprises three helices that form two separate anti-parallel coiled-coils and a loop that packs tightly against one of the coiled-coils (Figure 5a). This structure revealed a large positively charged surface that binds ZO-1 [58]. The cytoplasmic C-terminal coiled-coil region of occludin associates with mainly the GUK region of ZO-1 as shown by SAXS, NMR, and in vitro binding studies [59], which also revealed that serine phosphorylation within the acidic binding motif of the occludin coiled-coil significantly increases the binding affinity. Notably, several occludin isoforms result from alternative splicing and alternate promoter use, but neither this structural polymorphism nor the multitude of known post-translational modifications from proteolysis and serine, threonine or tyrosine phosphorylation of occludin [60] have so far been studied by X-ray or NMR methods. 

#### 2.1.4. Tricellulin

The precise definition of TJ architecture through freeze–fracture microscopy of epithelial preparations from rat intestine revealed a modified structure at tricellular junctions [61]. Tricellular pores and bicellular strand opening contribute to allowing the passage of large molecules through the TJ in the “leak pathway” as suggested by computational structural dynamics studies [62]. TJs completely disappear during the epithelial–mesenchymal transition (EMT), where the transcriptional repressor Snail plays a central role. The protein tricellulin was identified in a screen using Snail-overexpressing epithelial cells as a protein concentrated at tricellular tight junctions (tTJs) and named for this property [63]. 

Tricellulin is downregulated during the EMT. The E3 ubiquitin ligase Itch binds the N-terminus of tricellulin via its WW domain (named after two signature tryptophan residues) to stimulate its ubiquitination, which is, however, not primarily involved in proteasomal breakdown of tricellulin [64]. During apoptosis, cells are extruded from epithelial cell layers. Loss of functional tricellulin contributes to dissociation of tTJs during apoptosis, when it is cleaved by caspase at aspartate residues 441 and 487 in the C-terminal coiled-coil [65]. Tricellulin is of key importance for hearing, as it was reported that mutations in the human *TRIC* gene are associated with deafness [66].

Tricellulin is localized to tTJs but also to bicellular TJs. When tricellulin is selectively overexpressed at tTJs, it decreases the permeability for large solutes up to 10 kDa, but not for ions. This seemingly paradoxical observation may be explained by the rare occurrence of tricellular junctions relative to bicellular junctions [67]. Tricellular TJs are regarded as potential weak points in the paracellular barrier. Tricellulin-dependent macromolecular passage is observed in both leaky and tight epithelia [68]. Tricellulin tightens tricellular junctions and regulates bicellular TJ proteins. The extracellular loops of tricellulin may be crucial for its sealing function, because it could be shown that a synthetic peptide (trictide) derived from the tricellulin ECL2 may increase the passage of solutes into human adenocarcinoma cells [69]. In MDCK cells, the tricellulin C-terminus is important for basolateral translocation, whereas the N-terminus directs tricellulin to tricellular contacts. There is evidence for the formation of heteromeric tricellulin–occludin contacts at elongating bicellular junctions and of homomeric tricellulin–tricellulin complexes at tricellular junctions [70]. 

Tricellulin has an extended cytoplasmic N-terminus of 194 aa and a cytoplasmic C-terminal region of 195 aa, in marked contrast to occludin, where these regions include 66 aa and 256 aa, respectively. With the exception of the C-terminal coiled-coil domain, no cytoplasmic region carries a sequence signature suggesting a known domain structure in either protein. A crystal structure of the C-terminal coiled-coil domain of tricellulin was determined at 2.2-Å resolution (Figure 5b). This structure reveals a dimeric arrangement with an extended polar interface (Figure 5c), which may contribute to stabilizing tTJs [71].

#### 2.1.5. Other Tight-Junction Transmembrane Proteins

With the exception of tricellulin, the extracellular loops and ectodomains of the abovementioned transmembrane TJ proteins are involved in *trans* pairing interactions of opposing cells (Figure 1) [5]. For the POPDC and Crumbs family of transmembrane TJ proteins, no such cross–membrane interactions are described. The POPDC family of tri-span TM proteins consists of BVES/POPDC1, POPDC2, and POPDC3. BVES protein dimers are mediated by the cytoplasmic Popeye domain, and BVES–BVES *cis* pairing interactions are necessary to maintain epithelial integrity and junctional stability. The cytoplasmic tail of BVES was shown to directly interact with ZO-1 [14], but structural information on the atomic level is still missing [14]. Crumbs was first described in *D. melanogaster* [72]; in mammals it has three homologs (CRB1, CRB2, CRB3) of which the latter is expressed in all epithelial tissues [73]. As the Crumbs protein family members are part of the cell polarity complex Crumbs/PALS1/PATJ, further information is included in Section 2.2.3.

### 2.2. Proteins of the Cytoplasmic Plaque

**Figure 6:**
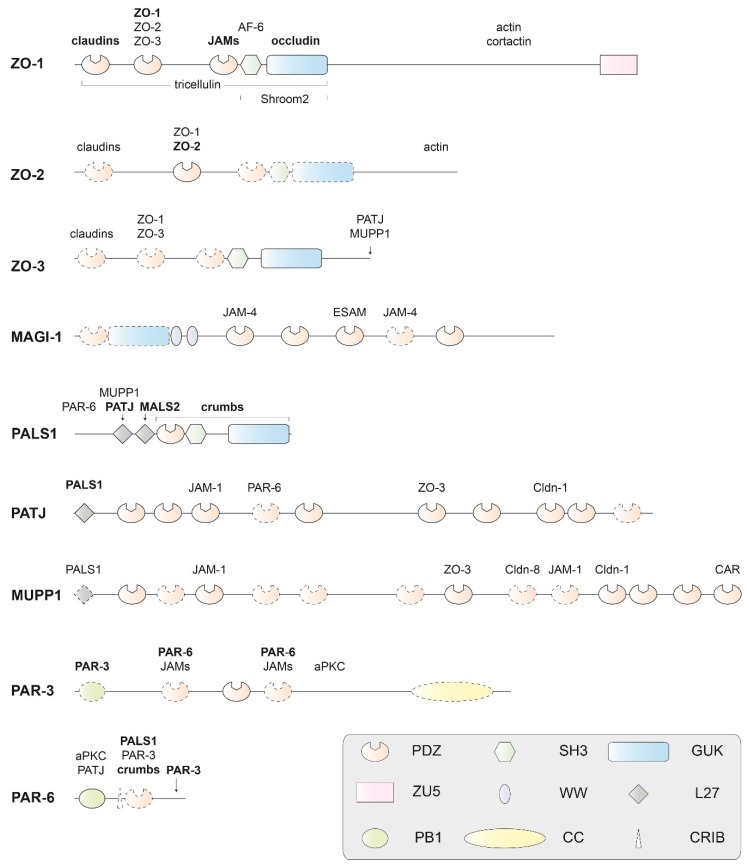
Domain structure of human PDZ domain-containing adapter proteins of the cytoplasmic plaque that interact physically with TM proteins of the tight junction. The proteins are scaled to the length of their amino-acid sequences. Experimental structures (usually by X-ray or NMR analysis) are available for protein domains drawn with solid contours, but not for domains drawn with dashed contours or for extended regions of polypeptide chains without domain annotation. Proteins binding to components of the human cytoplasmic plaque or their homologs are indicated above or below their interacting domains. With the exception of aPKC (as a subunit of the PAR-3/PAR-6/aPKC complex), only TM proteins or classical adapter proteins of the TJ are included as interacting proteins. Names of interacting proteins are written in bold letters, where the interaction is structurally characterized. Protein names and abbreviations are explained in the text or the legend of Figure 1. Domains are abbreviated as follows: PDZ: Initially identified in PSD-95 (postsynaptic density-95); DLG-1 (the *Drosophila* tumor suppressor protein discs large 1) and ZO-1; SH3: Src homology-3; PB1: Initially identified in PHOX and BEM1; ZU5: Present in ZO-1 and UNC5; L27: LIN-2/LIN-7; GUK: guanylate kinase homolog; WW: Named after two signature tryptophan residues; CC: coiled-coil; CRIB: CDC42/RAC interactive binding. Figure modified and updated after Guillemot et al. [3].

The proteins of the cytoplasmic plaque are characterized by recurrent protein–protein interaction (PPI) domains and frequently contain natively unfolded regions [74,75,76]. They are interconnected in a dynamic and multivalent PPI system, which has been partly mapped down to the domain level (Figure 6). In addition to the interactions displayed in the figure, there are multiple PPIs with regulatory and signaling proteins not covered in this review. 

#### 2.2.1. PDZ Domains

**Figure 7:**
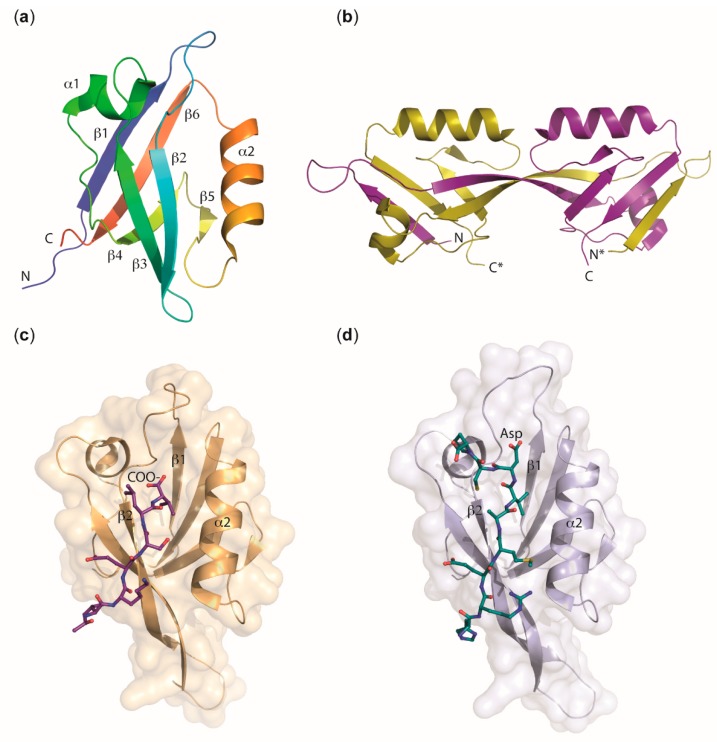
Structural features of PDZ domains. (**a**) Topology of a prototypical PDZ domain, here PALS 1 PDZ, PDB entry 4UU6 [87]. The polypeptide chain is drawn in rainbow colors changing from N- to C-terminus. A cleft for binding ligand peptides in an extended conformation is visible between strand β2 and helix α2. (**b**) Domain-swapped dimer formed by ZO-2 PDZ2, PDB entry 3E17 [88]. The two polypeptide chains are drawn in yellow and purple. The chain marked with an asterisk (*) corresponds to the second monomer within the dimer. Domain swapping moves the N-terminal β1 strand and half of β2 of one chain into the core structure of the other, leaving the ligand-binding geometry in both halves of the dimer intact. The PDZ2 domains in ZO-1, ZO-2, and ZO-3 are all found in the domain-swapped dimeric form [80,88,89,90,91]. (**c**) Canonical binding of a C-terminal ligand peptide to a PDZ domain, here PAR-6 PDZ bound to the hexapeptide VKRSLV, PDB entry 1RZX [92]. The terminal carboxy group is bound to the carboxylate-binding loop between strands β1 and β2 of PAR-6 PDZ and the extended ligand peptide aligns in antiparallel orientation with β2, extending the β-sheet. Note that this binding mode is energetically disfavored for PAR-6 PDZ and requires binding of CDC42 at a nearby CRIB domain (not shown). (**d**) Binding of an internal peptide to a PDZ domain, here PAR-6 PDZ bound to a dodecapeptide representing amino acids 29–40 of PALS1, PDB entry 1X8S [93]. The ligand peptide adopts an extended conformation with an aspartic acid side chain mimicking the carboxy group of the canonical C-terminal peptide ligand. Note the altered conformation of the carboxylate-binding loop.

Many proteins of the cytoplasmic plaque contain one or multiple PDZ domains (Figure 6). We next discuss some key features of these ubiquitous PDZ domains. PDZ domains regulate multiple cellular processes by promoting protein–protein interactions and are abundant protein modules in TJ proteins, but also in many other proteins in all kingdoms of life. Frequently, PDZ domains are associated with WW, SH2, SH3 (Src homology 2 or 3), or PH (Pleckstrin homology) domains within one polypeptide chain [77]. The term PDZ is derived from the three founding members of the family, PSD-95 (postsynaptic density-95), the *Drosophila* tumor suppressor protein DLG-1 (discs large 1), and ZO-1. As early as 2010, > 900 PDZ domains were annotated in > 300 proteins encoded in the mouse genome, and > 200 X-ray or NMR structures of PDZ domains from various sources were known [78]. In August 2019, a PDB [79] search returned 533 entries with the keyword “PDZ domain” and 138 entries with the keyword “PDZ domain-like”. Thus, extensive structural data are available for these domains. In general, PDZ domains are structured as a β-sandwich capped by two α-helices and bind ligand peptides in a shallow groove between helix α2 and strand β2 (Figure 7a). Their propensity to dimerize via domain swapping was first described for the second PDZ domain (PDZ2) of ZO-2 [80] and later also for PDZ2 of ZO-1 and ZO-3 (Figure 7b, see Section 2.2.2.). 

Domain swapping is frequently observed in small β-sheet domains. Bacterial major cold-shock proteins [81,82], for example, were found to form domain-swapped dimers. A domain-swapped three-stranded segment of the *E. coli* cold-shock protein CspA is capable of recombining with a polypeptide region of ribosomal protein S1 to form a closed β-barrel recapitulating structural features of both parent proteins [73].

PDZ domains have been divided into three specificity classes according to the preferred amino acid residue at position –2 (P^−2^) of the binding groove [72]. Typically, PDZ domains recognize sequence motifs at the extreme carboxy terminus of ligand proteins (Figure 7c), but binding of internal sequence motifs is also common (Figure 7d). 

PDZ domains are regarded as promising drug targets for neurological and oncological disorders, as well as viral infections. Many structure-guided efforts are underway towards the development of small-molecule or peptidic modulators of PDZ domains [83,84], including the PDZ domains from Shank3, a central scaffolding protein of the post-synaptic density protein complex [85] and of the protein interacting with C kinase (PICK1), a regulator of AMPA receptor trafficking at neuronal synapses [86].

#### 2.2.2. MAGUK Proteins

Membrane-associated guanylate kinase homologs (MAGUKs) constitute a family of scaffolding molecules with a core MAGUK module consisting of a PDZ, SH3, and an enzymatically inactive guanylate kinase (GUK) domain [94,95,96]. The MAGUK protein family members ZO-1, ZO-2, and ZO-3 link the TJ membrane proteins to the cytoskeleton and provide the structural basis for the assembly of multiprotein complexes at the cytoplasmic side of TJs (Figure 1) [97]. ZO-1 is a cytoplasmic component of both TJs and AJs, and connects the TJ to the actin cytoskeleton via extended, presumably unstructured polypeptide regions near its C-terminus [98]. Direct actin binding was also reported for ZO-2 and ZO-3 [24,54,55].

ZO-1 and its paralogs ZO-2 and ZO-3 contain three N-terminal PDZ domains (Figure 6). The propensity of these PDZ domains to recognize specific C-terminal or other peptide motifs and assemble multicomponent TJ protein complexes will be highlighted below. Most of the claudins present at TJs have conserved C-terminal tails that bind to PDZ1 of ZO proteins. Compared to the single PDZ domain of the AJ protein Erbin, the ZO-1 PDZ1 domain has a broadened ligand specificity. Crystal structures of the Erbin PDZ and the ZO-1 PDZ1 revealed the structural basis for the different ligand specificities, where subtle conformational rearrangements are identified at multiple ligand-binding subsites, and support a model for ligand recognition by these domains [99]. 

The intracellular C-terminus of claudins binds to the N-terminal PDZ1 domain of ZO proteins with variable affinity. The affinity of claudin binding to ZO-1 PDZ1 depends on the absence or presence of a tyrosine residue at position -6 from the claudin C-terminus. Crystal structures of ZO-1 PDZ1 with empty ligand-binding groove, with a bound claudin-1 heptapeptide, which does not have a Tyr -6, or with a bound claudin-2 heptapeptide containing a Tyr -6 revealed significantly different binding geometries explaining the influence of the signature tyrosine residue on binding affinity [100]. In addition to claudin binding, the ZO-1 PDZ1 also mediates interactions with phosphoinositides. Mapping the inositol hexaphosphate binding site onto an NMR structure of ZO-1 PDZ1 revealed spatial overlap with the claudin binding surface and thus provided a structural rationale for the observed competition of both ligands for ZO-1 [101]. 

The second PDZ domain (PDZ2) of ZO-proteins is known to promote protein dimerization [24,54,55]. A crystal structure shows that ZO-1—PDZ2 dimerization is stabilized by extensive domain swapping of β-strands. This structural rearrangement leaves the canonical peptide-binding groove intact in both subunits of PDZ2 dimer, which are composed of elements from both monomers [89]. Domain swapping of human ZO-1 PDZ2 was subsequently confirmed by solution NMR analysis. In this study, the importance of strand β2 for the domain exchange was demonstrated by insertion mutagenesis [91]. NMR analysis clearly demonstrated that PDZ2 of ZO-2 may also dimerize by domain swapping. A 1.75 Å resolution crystal structure of the ZO-2 PDZ2 confirms formation of a domain-swapped dimer with exchange of β-strands 1 and 2 (Figure 7b) [88], and there is evidence for the formation of PDZ2-promoted domain-swapped homodimers in all three ZO proteins. Based on this observation and the high sequence similarity between the ZO-1, -2, and -3 PDZ2 domains (66% sequence identity between ZO-1 and ZO-2, 50% between ZO-1 and ZO-3, 54% between ZO-2 and ZO-3), heterodimer formation between them was proposed as a potential mechanism of forming and stabilizing the cytoplasmic plaque [80]. Structural evidence for domain-swapped heterodimers of ZO proteins is, however, still lacking. 

ZO-1 PDZ2 interacts with connexins, in particular the abundant connexin43, which functions in gap junction formation and regulation. X-ray and NMR analyses showed that domain swapping of ZO-1 PDZ2 preserves the carboxylate tail-binding pockets of the PDZ domains and creates a distinct interface for connexin43 binding [90]. 

The third PDZ domain (PDZ3) of ZO proteins is important for the interaction with the C-terminus of transmembrane JAMs. A crystal structure of ZO-1 PDZ3 was determined at 1.45 Å resolution. This study established that ZO-1 PDZ3 preferentially binds ligands of sequence type X[D/E]XΦ_COOH_ where X may be any amino acid and Φ is a hydrophobic residue [72].

Following the three N-terminal PDZ domains, ZO proteins contain a SH3-GUK module. Crystal structure analysis of the ZO-1 SH3-GUK tandem domain confirmed independent folding of the SH3 and GUK domains, and pulldown assays identified the downstream U6 loop as an intramolecular ligand of the SH3-GUK core with a potential role in regulating TJ assembly in vivo [102]. Crystal structures of the complete MAGUK core module of ZO-1 comprising the PDZ3-SH3-GUK region and its complex with the cytoplasmic tail of adhesion molecule JAM-A revealed that residues from the adjacent SH3 domain are involved in ligand binding to the ZO-1 PDZ3 [103].

ZO-1 is distinct from its paralogs ZO-2 and ZO-3 by the presence of an extended C-terminal region harboring a ZU5 domain [104,105] and described to mediate physical interaction with the CDC42 effector kinase MRCKβ. An NMR structure showed the ZO-1 ZU5 domain to adopt a β-barrel structure, which is incomplete in comparison with homologous proteins by lacking two β-strands. Attempts to analyze the structure of a ZO-1 ZU5/MRCKβ complex remained unsuccessful, but evidence could be provided that GRINL1A (glutamate receptor, ionotropic, N-methyl-D-aspartate-like 1A combined protein) binds ZO-1 ZU5 in a very similar way as MRCKβ. NMR analysis then showed that a 22-aa GRINL1A peptide hairpin associates with the ZO-1 ZU5 domain to form a complete canonical ZU5 domain [106].

In the MAGI proteins (MAGUKs with inverted domain structure), the characteristic arrangement of PPI domains present in common MAGUK proteins is inverted. Furthermore, the MAGI proteins contain two WW domains in place of the SH3 domain found in MAGUKs (Figure 6) [107]. The family member MAGI-1 is tethered to TJs through interactions of its PDZ domains with the C-terminus of the non-classical junctional adhesion molecule JAM-4 [108]. In addition to its function in the TJ, the first PDZ domain of MAGI-1 binds peptide ligands derived from the oncoprotein E6 of human papillomavirus and the ribosomal S6 kinase 1 (RSK1) [109,110]. NMR analysis suggests the involvement of peptide regions flanking the PDZ domain in ligand binding [109]. PALS1 (protein associated with Lin seven 1, also known as MPP5—membrane-associated palmitoylated protein 5) is another member of the MAGUK family (Figure 6) and described below as part of the Crumbs/ PALS1/PATJ complex.

#### 2.2.3. The Crumbs/PALS1/PATJ Complex

The Crumbs/PALS1/PATJ complex is involved in establishing and maintaining cell polarity and located in the cytoplasmic plaque of TJs [2,3,111]. Crumbs is a single-span TM protein, whereas the other proteins present in this complex, PALS1 and PATJ (PALS-associated tight-junction protein), are cytoplasmic scaffolding proteins (Figure 1). PALS1 functions as an adaptor protein mediating indirect interactions between Crumbs and PATJ (Figure 6). Both PALS1 and PATJ share an N-terminal L27 domain. L27 domains organize scaffold proteins into supramolecular complexes by heteromeric L27 interactions. PATJ is recruited to TJs through interactions with the C-termini of claudin-1 and ZO-3 [112].

The PATJ–PALS1 interaction is mediated by the single L27 domain of PATJ and the N-terminal L27 domain (L27N) of PALS1 [113]. A crystal structure of the PALS1-L27N/PATJ-L27 heterodimer shows that each L27 domain is composed of three α-helices and that heterodimer formation is due to formation of a four-helix bundle by the first two α-helices of the L27 domains and coiled–coil interactions between the helices α3 [114]. NMR structure analysis revealed closely similar topologies for heterotetrameric mLin-2/mLin-7 and PATJ/PALS1 complexes, suggesting a general assembly mode for L27 domains [115]. A crystal structure of a heterotrimeric complex formed by the N-terminal L27 domain of PATJ, the N-terminal tandem L27 domains of PALS1, and the N-terminal L27 domain of MALS2 (mammalian homolog-2 of Lin-7) revealed an assembly of two cognate pairs of heterodimeric L27 domains. This structure is thought to reveal a novel mechanism for tandem L27 domain-mediated supramolecular complex assembly [116].

The intracellular functions of Crumbs3 (CRB3) are mediated by its conserved 37-aa cytoplasmic tail (Crb-CT) and its interaction with PALS1 and the actin-binding protein moesin. The crystal structure of a PALS1 PDZ-SH3-GUK/Crb-CT complex shows that all three domains of PALS1 contribute to Crb-CT binding [117]. A further crystal structure of human PALS1 PDZ bound to 17-aa C-terminal CRB1 peptide shows that only the very C-terminal tetrapeptide ERLI is involved in direct binding to PALS1 PDZ. Comparison with apo-PALS1 PDZ (Figure 7a) revealed that a key phenylalanine residue in the PALS1 PDZ controls access to the ligand-binding groove [87]. To reveal the nature of the Crumbs/moesin interaction, the FERM (protein 4.1/ezrin/radixin/moesin) domain of murine moesin was co-crystallized with the soluble C-terminus of *Drosophila* Crumbs. The 1.5-Å resolution crystal structure revealed that both the FERM-binding motif, as well as the PDZ-binding motif present in the Crumbs C-terminal peptide contribute to the interaction with moesin. Phosphorylation of the Crb-CT by atypical protein kinase C (aPKC) disrupts the Crumbs/moesin association but not the Crumbs/PALS1 interaction. Crumbs may therefore act as aPKC-mediated sensor in epithelial tissues [118]. 

#### 2.2.4. The PAR-3/PAR-6/aPKC Complex

Similar as the Crumbs/PALS1/PATJ complex, the evolutionarily conserved PAR-3/PAR-6/aPKC complex is associated to TJs and crucial for establishing and maintaining cell polarity. The complex formed by the PAR (partition defective)-3 and PAR-6 proteins, as well as the atypical protein kinase C (aPKC) interacts with subunits from the Crumbs/PALS1/PATJ complex and is regulated by binding to the small GTPases CDC42 and RAC1. Composition and stoichiometry of the PAR-3/PAR-6/aPKC complex are linked to cell polarity and to the cell cycle [119].

Human PAR-6 contains a single PDZ domain, which mediates binding to the C-terminus of TM receptor CRB3. Binding of C-terminal ligands to the PAR-6 PDZ depends on binding of the Rho-GTPase CDC42 to a CRIB domain adjacent to the PAR-6 PDZ. In addition, the PAR-6 PDZ also binds internal peptides, e.g., from PALS1 and its *Drosophila* homolog Stardust. The regulation of ligand binding to PAR-6 PDZ by CDC42 has been structurally characterized in a number of studies. A 2.5-Å crystal structure of a PAR-6 PDZ-bound internal dodecapeptide derived from PALS1 revealed a characteristic deformation of the carboxylate-binding loop of PAR-6 PDZ relative to the structure with bound C-terminal ligand (Figure 7d) [93]. The structural adjustments associated with regulator and ligand binding to the PAR-6 PDZ were also highlighted in a 2.1-Å crystal structure and an NMR structure of the PAR-6 PDZ domain (Figure 7c), which revealed deviations from the canonical PDZ conformation that account for low-affinity binding of C-terminal ligands. CDC42 binding to the adjacent CRIB domain triggered a structural transition to the canonical PDZ conformation and was associated with a ~13-fold increase in affinity for C-terminal ligands [92]. NMR structures of the isolated PAR-6 PDZ domain and a disulfide-stabilized CRIB-PDZ fragment identified a conformational switch in the PAR-6 PDZ domain that is linked to the increase in ligand affinity induced by CDC42 binding to PAR-6 [120]. Finally, NMR analysis of a C-terminal Crumbs peptide binding to PAR-6 and the crystal structure of the PAR-6 PDZ/peptide complex indicated why the affinity of this interaction is 6-fold higher than in previously studied PAR-6/peptide binding studies [121].

PAR-3 acts as central organizer of the PAR-3/PAR-6/aPKC complex and is thus essential for establishment and maintenance of cell polarity. In *Caenorhabditis elegans*, PAR-3 mediates TJ binding through interaction with junctional adhesion molecule (JAM) [122,123]. In cultured endothelial cells, PAR-3 associates with JAM-2 and JAM-3, but neither with the related Ig-like TM proteins ESAM nor CAR [124]. PAR-3 contains an N-terminal oligomerization domain in addition to three PDZ domains. NMR analysis showed the monomeric PAR-3 N-terminal domain (NTD) to adopt a PB1-like fold and to oligomerize into helical filaments. This interaction was proposed to facilitate the assembly of higher-order PAR-3/PAR-6/aPKC complexes [125]. The ability of the PAR-3 NTD to self-associate and form filamentous structures was further studied by crystallographic analysis of the PAR-3 NTD and analysis of the filament structure by cryo-electron microscopy (cryo-EM). Here, it was revealed that both lateral and longitudinal packing within PAR-3 NTD filaments is primarily mediated by Coulomb interactions [126].

The second PDZ domain of PAR-3 binds phosphatidylinositol (PI) lipid membranes with high affinity as shown in a biochemical and NMR study of PAR-3 PDZ2. This study also showed that the lipid phosphatase PTEN (phosphatase and tensin homolog) binds PAR-3 PDZ3 and thus cooperates with PI in regulating cell polarity through PAR-3 [127]. A three-dimensional structure of the second PDZ domain of human PAR-3 was also determined as part of an NMR structure analysis automation study [128]. A previously unknown C-terminal PDZ-binding motif, identified in PAR-6 through crystal structures and NMR binding analyses, mediates interactions with PDZ1 and PDZ3, but not with PDZ2 of PAR-3. Evidently, PAR-3 has the ability to recruit two PAR-6 molecules simultaneously, possibly facilitating the assembly of polarity protein networks through these interactions [129]. 

#### 2.2.5. Other Cytoplasmic Tight-Junction Proteins

In addition to the MAGUK proteins, the subunits and regulators of the Crumbs/PALS1/PATJ and PAR-3/PAR-6/aPKC complexes, various other cytoplasmic proteins are associated with TJs. The multiple PDZ domain protein 1 (MUPP1, also known as MPDZ) contains 13 PDZ domains. MUPP1 is a paralog of PATJ, shares several TJ-binding partners (Figure 6) and a similar subcellular localization, but displays a distinct selectivity in its interactions with claudins and is dispensable for TJ formation while PATJ is not [130,131]. A crystal structure of the twelfth PDZ domain of MUPP1 was determined in a structural genomics effort to crystallize PDZ domains with self-binding C-terminal extensions [132]. Several additional MUPP1-PDZ domains were analyzed within the research program of the Center for Eukaryotic Structural Genomics [133] and submitted to the Protein Data Bank (PDB) [79], but without functional annotation. Crystal structure analysis of the mouse MUPP1-PDZ4 domain revealed a canonical PDZ fold with six β-strands and three α-helices [60]. The angiomotin (AMOT) proteins [134] were reported to interact with MUPP1, PATJ [135], ZO-1 and MAGI-1b, and ascribed a role in the assembly of endothelial cell junctions [25]. The cytoskeletal linker cingulin, a predicted dimeric coiled-coil protein of unknown three-dimensional structure, was initially characterized as a peripheral TJ component [136]. Although its amino-terminal region was reported to interact with ZO-1 in cells [137], cingulin was later shown to be dispensable for TJ integrity and epithelial barrier function [138]. The ZO-1 associated nucleic acid-binding protein ZONAB (also referred to as YBX3 or CSDA1) is a transcription factor that shuttles between TJs and the nucleus and regulates epithelial cell proliferation [139,140]. Although a crystal or NMR structure of ZONAB is not available, the conformation and nucleic acid binding of its N-terminal cold shock domain may be inferred from structures of bacterial major cold shock proteins [141] or the homologous Y-box factor YB-1 [142]. 

The molecular composition of TJs varies significantly between different epithelia and determines their dual functions as effective barriers for solutes or channels for particular classes of solutes [143]. Therefore, the expression patterns of TJ proteins in various tissues are kept under tight control by various transcription factors in addition to ZONAB. A large number of TJ and AJ transmembrane (TM) proteins are under transcriptional control by the Grainyhead-like proteins GRHL1 and GRHL2 or by nuclear receptors [144]. These transcription factors therefore regulate a large subset of proteins, making up the apical junction complex. Frequently, one transcription factor controls the expression of multiple genes encoding TJ or AJ proteins. For example, GRHL2 acts as transcriptional activator of both AJ and TJ components including several claudins and thus functions as regulator of epithelial differentiation [145]. Equally frequently, one TJ protein is controlled by multiple transcription factors. The *Cldn4* gene, being under transcriptional control by GRHL2, GRHL3, the androgen receptor, retinoic acid/retinoid X receptors, and p63 in different tissues [144,146,147], serves as an impressive example here. Although structural information is available for several of these factors [148,149,150,151], these proteins will not be further discussed here, where the focus is placed on proteins of the TJ core structure. Equally, the large set of signaling and effector proteins acting on the TJ [2,11,12] will not be discussed further.

## 3. Conclusions

Here, we have reviewed three-dimensional structures of TJ proteins, focusing on the core TJ complex. It becomes clear that our knowledge of these structures is fairly incomplete, because most TJ proteins do not lend themselves easily to structure analysis due to their size and/or the presence of TM regions, natively unfolded polypeptide segments or heterogeneous post-translational modifications [74,75,76]. Our knowledge of protein–protein interactions within the TJ also does not go far beyond structural features of selected binary interactions, often involving small protein fragments or peptides. At a resolution that permits construction of atomic models, very little is known about the general architecture of the TJ. Herein lies a great challenge and opportunity for future research making use of new integrative methods in structural biology, including cryo-electron microscopy [152,153,154,155], cryo-electron tomography [156,157], cross-linking mass spectrometry [158,159], small-angle X-ray and neutron scattering [160,161], and others [162]. These rapidly emerging and developing methods can be informed by the available high-resolution structures of TJ proteins, protein domains and protein–protein interactions. We may expect to see exciting results along these lines in the near future, revealing the architecture of TJs at high resolution in defined functional states.

## Abbreviations

aa	Amino acid
AJ	Adherens junction
BBB	Blood-brain barrier
CTD	C-terminal domain
ECL	Extracellular loop
EMT	Epithelial-mesenchymal transition
NTD	N-terminal domain
PDB	Protein Data Bank
PPI	Protein–protein interaction
TJ	Tight junction
tTJ	Tricellular tight junction
TM	Transmembrane
